# Healthier central England or North–South divide? Analysis of national survey data on smoking and high-risk drinking

**DOI:** 10.1136/bmjopen-2016-014210

**Published:** 2017-03-01

**Authors:** Emma Beard, Jamie Brown, Robert West, Colin Angus, Eileen Kaner, Susan Michie

**Affiliations:** 1 Research Department of Clinical, Educational and Health Psychology, University College London, London, UK; 2 Department of Epidemiology and Public Health, University College London, London, UK; 3 ScHARR, School of Health and Related Research, University of Sheffield, Sheffield, UK; 4 Institute of Health and Society, Newcastle University, Newcastle, UK

**Keywords:** Alcohol, Tobacco, Regional

## Abstract

**Objectives:**

This paper compares patterns of smoking and high-risk alcohol use across regions in England, and assesses the impact on these of adjusting for sociodemographic characteristics.

**Design:**

Population survey of 53 922 adults in England aged 16+ taking part in the Alcohol and Smoking Toolkit Studies.

**Measures:**

Participants answered questions regarding their socioeconomic status (SES), gender, age, ethnicity, Government Office Region, smoking status and completed the Alcohol Use Disorders Identification Test (AUDIT). High-risk drinkers were defined as those with a score of 8 or more (7 or more for women) on the AUDIT.

**Results:**

In unadjusted analyses, relative to the South West, those in the North of England were more likely to smoke, while those from the East of England, South East and London were less likely. After adjustment for sociodemographics, smoking prevalence was no higher in North East (RR 0.97, p>0.05), North West (RR 0.98, p>0.05) or Yorkshire and the Humber (RR 1.03, p>0.05) but was less common in the East and West Midlands (RR 0.86, p<0.001; RR 0.91, p<0.05), East of England (RR 0.86, p<0.001), South East (RR 0.92, p<0.05) and London (RR 0.85, p<0.001). High-risk drinking was more common in the North but was less common in the Midlands, London and East of England. Adjustment for sociodemographics had little effect. There was a higher prevalence in the North East (RR 1.67, p<0.001), North West (RR 1.42, p<0.001) and Yorkshire and the Humber (RR 1.35, p<0.001); lower prevalence in the East Midlands (RR 0.69, p<0.001), West Midlands (RR 0.77, p<0.001), East of England (RR 0.72, p<0.001) and London (RR 0.71, p<0.001); and a similar prevalence in the South East (RR 1.10, p>0.05)

**Conclusions:**

In adjusted analyses, smoking and high-risk drinking appear less common in ‘central England’ than in the rest of the country. Regional differences in smoking, but not those in high-risk drinking, appear to be explained to some extent by sociodemographic disparities.

**Strengths and limitations of this study**

- Used a representative survey about smoking and drinking conducted on a large sample of the adult population in England.
- Based on the most up-to-date information in England on regional differences in smoking and high-risk drinking accounting for disparities in gender, socioeconomic status (SES), ethnicity and age.
- Respondents may have underestimated or failed to report their drinking and smoking.
- Patterns of smoking and alcohol use were only available at the Government Office Region level, whereas important variation may occur at a more micro-geographical level.

## Introduction

In England, around 20% of the population are smokers and 13% drink excessively.^1^ These behaviours are leading risk factors for several non-communicable diseases, including cancer, diabetes and chronic respiratory and cardiovascular conditions.^2–4^ It is estimated that around 8000 deaths/year are alcohol-related^5^ and 80 000 deaths of adults aged 35 and over are attributed to smoking annually.^6^ The prevalence and adverse effects of high-risk drinking and tobacco use are not equally distributed across the country, with large regional variations.^7–10^ A North–South divide exists for smoking, with higher rates of tobacco use, smoking-related deaths and smoking-related harm in northern regions.^10^ ^11^ In contrast, excessive alcohol consumption tends to be lowest in central and eastern regions, while an East versus West divide is seen in the prevalence of alcohol dependency and alcohol sales.^12^ ^13^ These regional variations in consumption do not always map onto experienced harm, a phenomenon known as the Alcohol Harm Paradox.^14^ In 2014, alcohol-related death rates were significantly higher among regions in the north of England compared with those in the south.^15–17^

The 2009 Commission on the Social Determinants of Health, set up by the World Health Organization (WHO) called for a greater understanding of what may account for these within-country inequalities and for the assessment of the full impact of the problem.^18^ The UK government has also set performance targets for reducing geographical inequalities in health.^19^ One proposed factor is disparities in sociodemographic profiles,^20^ ^21^ with southern regions having smaller differences in sex ratios,^22^ a larger proportion of residents in managerial and professional occupations,^22^ ^23^ and an older population^24^ than other regions; while the proportion of ethnic minority groups is greatest in the Midlands and London.^25^ Rates of drinking above guideline levels have been shown to increase with age^26^ and are lowest among more disadvantaged groups (ie, women, ethnic minorities and those of lower socioeconomic status (SES)).^27^ In contrast, alcohol-related harm rises sharply with decreasing SES.^27^ Self-reported cigarette smoking is greater among most ethnic minority groups (14% of the population), particularly those from Pakistani and Bangladeshi backgrounds, and peaks between the age of 24 and 34.^10^ There is also a clear socioeconomic divide, with a higher prevalence of smoking among the most disadvantaged.^28^ ^29^

Only a handful of publications until now have looked at this level of granularity.^30^ Data from the Health Survey for England showed the highest prevalence of ‘binge drinking’ in the North and lowest prevalence in the South West.^12^ After adjustment for individual and area-based sociodemographic characteristics, prevalence was on average greater in the North East and North West, while London had the lowest rates of high-intensity drinking. The pattern is somewhat different for frequency of drinking, with those in Southern regions more likely to report that they drink on most days.^31^ Data from the British Health and Lifestyle Survey noted that individual characteristics have an independent effect on neighbourhood variations in smoking but that significant between-ward differences in smoking behaviour remain which cannot be explained either by population composition or ward-level deprivation.^32^ However, there are several issues with these previous studies. Some failed to adjust for the full range of sociodemographic variables which may explain, at least in part, regional variations (eg, ethnicity), while others failed to consider the moderation effects of these sociodemographic variables.^27^

Given these limitations, evidence for the temporal instability of regional variations,^12^ and that the North–South divide may be increasing;^33^ ^34^ there is a need for up-to-date prevalence statistics on regional variations in excessive alcohol consumption and smoking, adjusting for a range of sociodemographic characteristics. Thus, this paper aims to provide up-to-date prevalence statistics on smoking and high-risk alcohol use across different regions in England, and to assess whether sociodemographic composition (e.g., age, gender, ethnicity and SES) may account for any disparities. A secondary aim was to assess whether regional patterns were similar across sociodemographic subgroups by looking at moderation effects. Such findings could help to provide clues as to how best to address these behaviours at national and regional levels.

Data were used from the Alcohol Toolkit (ATS) and Smoking Toolkit (STS) studies,^35^ ^36^ which are household population surveys of adults aged 16+. These surveys have advantages over other population surveys, including monthly data collection, speed of data release, use of a validated measure of alcohol consumption—Alcohol Use Disorders Identification Test (AUDIT)^37^ ^38^—and large sample size.^35^ They also afford the ability, unlike previous studies, to make comparisons between smoking and alcohol use, given that the same participants complete both surveys.

## Methodology

### Design

Data were from the ATS and STS between March 2014 and October 2016. The ATS and STS involve monthly cross-sectional household computer-assisted interviews, conducted by Ipsos Mori, of ∼1700 adults aged 16+ in England. The baseline survey uses a type of random location sampling, which is a hybrid between random probability and simple quota sampling (see http://www.smokinginengland.info and http://www.alcoholinengland.info or the published protocols^35^ ^36^ for more details).

### Ethical approval

Ethical approval for the Smoking Toolkit Study (STS) was originally granted by the UCL Ethics Committee (ID 0498/001). Approval for the ATS was granted by the same committee as an extension of the STS. The data are not collected by UCL and are anonymised when received by UCL. Explicit verbal agreement and willingness to answer questions voluntarily is recorded electrically by Ipsos Mori, the company administering the survey. This is standard protocol and was agreed by the UCL ethics committee. Participants are also given a printed information sheet.

### Study population

Data were collected on 53 922 adults aged 16 and over in England. Table 1 shows the characteristics of participants overall and as a function of their smoking and high-risk drinking status. Five per cent (95% CI 4.2% to 5.9%) of participants resided in the North East, 13.3% (95% CI 12.5% to 14.1%) in the North West; 10.2% (95% CI 9.4% to 11.0%) in Yorkshire and the Humber, 8.7% (95% CI% 7.9 to 9.5%) in the East Midlands, 10.2% (95% CI 9.4% to 11.0%) in the West Midlands, 11.2% (95% CI 10.5% to 12.0%) in the East of England, 14.8% (95% CI 14.0% to 15.6%) in Greater London Authority, 16.2% (95% CI 15.5% to 17.0%) in the South East and 10.3% (95% CI 9.5% to 11.1%) in the South West.

**Table 1:** Characteristics of participants overall and as a function of their smoking and high-risk drinking status

	Overall (n=53 922)			Smoker (n=9999)			Non-smoker (n=43 923)			High-risk drinker (n=7869)			Non-high-risk drinker and non-drinkers (n=46 053)		
	Per cent	95% CI		Per cent	95% CI		Per cent	95% CI		Per cent	95% CI		Per cent	95% CI	
		Lower	Upper		Lower	Upper		Lower	Upper		Lower	Upper		Lower	Upper
Female	51.1	50.3	51.8	51.8	51.2	52.5	47.5	46.0	48.9	39.1	37.4	40.8	53.0	52.4	53.7
Age															
16–24	14.3	13.6	15.1	18.1	16.4	19.9	13.5	12.6	14.3	23.8	21.9	25.7	12.7	11.9	13.6
25–34	16.7	16.0	17.5	22.0	20.3	23.7	15.5	14.7	16.4	17.7	15.7	19.7	16.6	15.7	17.4
35–44	16.7	15.9	17.5	18.7	17.0	20.5	16.2	15.4	17.1	17.3	15.3	19.3	16.6	15.7	17.4
45–54	17.4	16.6	18.1	18.3	16.5	20.1	17.2	16.3	18.0	19.8	17.8	21.8	17.0	16.1	17.8
55–64	14.0	13.3	14.8	12.6	10.8	14.4	14.4	13.5	15.2	12.7	10.6	14.8	14.3	13.4	15.1
65+	20.8	20.1	21.6	10.2	8.4	12.1	23.2	22.4	24.1	8.7	6.6	10.8	22.9	22.1	23.7
Ethnicity															
White	86.1	85.8	86.4	90.5	89.8	91.1	85.1	84.8	85.5	96.3	95.9	96.8	84.4	84.0	84.7
Mixed/multiple ethnic group	1.2	0.4	2.0	1.4	0.0	3.4	1.1	0.2	2.1	1.2	0.0	3.4	1.2	0.3	2.1
Asian or British Asian	8.1	7.3	8.9	4.8	2.9	6.7	8.9	8.0	9.8	0.9	0.00	3.1	9.3	8.5	10.2
Black African/Caribbean/Black British	3.1	2.2	3.9	1.7	0.0	3.6	3.4	2.5	4.3	0.8	0.00	3.0	3.5	2.6	4.4
Other ethnic group	1.5	0.7	2.3	1.6	0.0	3.6	1.5	0.6	2.4	0.8	0.00	3.0	1.6	0.7	2.5
Social grade															
AB	26.6	25.8	27.3	14.0	12.1	15.8	30.0	29.2	30.8	29.8	27.9	31.6	26.6	25.8	27.3
C1	27.0	26.2	27.8	23.5	21.8	25.2	28.3	27.5	29.1	30.0	28.2	31.9	27.0	26.2	27.8
C2	22.0	21.2	22.8	26.4	24.8	28.1	21.0	20.1	21.8	22.1	20.2	24.1	22.0	21.2	22.8
D	15.8	14.9	16.6	20.8	19.0	22.5	13.8	12.9	14.6	10.9	8.8	13.0	15.8	14.9	16.6
E	8.7	7.8	9.6	15.3	13.5	17.1	6.9	6.0	7.8	7.2	5.0	9.3	8.7	7.8	9.6
Education															
University education	30.7	30.0	31.4	17.7	15.9	19.5	33.7	32.9	34.4	32.0	30.1	33.8	30.5	29.7	31.2
A-level and equivalent	18.7	17.9	19.4	19.1	17.3	20.9	18.6	17.7	19.4	26.3	24.4	28.2	17.4	16.6	18.2
GCSE/vocational	28.4	27.7	29.1	36.8	35.3	38.4	26.5	25.6	27.3	27.9	26.0	29.7	28.5	27.7	29.2
Other/still studying	7.5	6.7	8.3	6.6	4.7	8.4	7.7	6.8	8.6	6.4	4.3	8.6	7.7	6.8	8.6
None	14.7	13.9	15.5	19.8	18.0	21.5	13.6	12.7	14.4	7.5	5.3	9.6	16.0	15.1	16.8
Income															
£40 000+	31.0	30.3	31.8	22.6	20.9	24.4	33.0	32.1	33.8	18.1	18.1	18.1	29.6	28.7	30.4
£17 500 to £3999	32.5	31.7	33.2	31.5	29.7	33.3	32.7	31.8	33.5	18.1	18.1	18.1	32.7	31.9	33.6
£9500 to £17 499	18.6	17.8	19.3	20.6	18.8	22.5	18.1	17.2	19.0	18.1	18.1	18.1	19.5	18.6	20.3
<£11 499	17.9	17.2	18.7	25.2	23.4	27.0	16.3	15.4	17.2	18.1	18.1	18.1	18.3	17.4	19.1
In full-time employment	48.6	47.9	49.3	48.2	46.8	49.6	47.3	46.6	48.0	40.9	39.2	42.6	48.6	47.9	49.3
Composite SES score															
1st quartile (high)	29.9	29.2	30.6	16.1	14.3	17.9	33.0	32.2	33.8	35.0	33.2	36.8	29.0	28.2	29.8
2nd quartile	24.2	23.5	25.0	22.1	20.4	23.9	24.7	23.9	25.6	27.1	25.1	29.0	23.8	22.9	24.6
3rd quartile	24.6	23.8	25.3	29.4	27.7	31.1	23.5	22.6	24.3	24.0	22.0	26.0	24.7	23.8	25.5
4th quartile (low)	21.3	20.6	22.1	32.3	30.7	34.0	18.8	18.0	19.7	14.0	11.9	16.0	22.6	21.8	23.4

### Measures

Age, gender, Government Office Region (London, South East, South West, East Anglia, East Midlands, West Midlands, Yorkshire/Humberside, North West and North East), ethnicity and SES were measured by the ATS and STS.

Participants were grouped into five ethnic categories according to the question adopted by the 2011 England and Wales census:^39^ White (White British, White Irish, White Gypsy/Traveller, White other); Mixed/Multiple ethnic group (Mixed White/Black Caribbean, White/Black African, White/Asian, White other); Asian or British Asian (Asian Indian, Asian Pakistani, Asian Bangladeshi, Asian Chinese, Asian other); Black African/Caribbean/Black British (Black African, Black Caribbean, Black other); Other ethnic group (Arab, other).

SES was measured using four measures detailed below: social grade, annual income, educational level and working status. These measures have all been associated with alcohol and smoking behaviour, and combined should reflect the multifaceted nature of SES.^27–29^

1. Social grade: measured using the British National Readership Survey (NRS) Social-Grade Classification Tool:^40^ A: higher managerial, administrative or professional; B: intermediate managerial, administrative or professional; C1: supervisory or clerical and junior managerial administrative or professional; C2: skilled manual workers; D: semiskilled and unskilled manual workers; E: causal or lowest grade workers, pensioners and others who depend on the welfare state for their income.
2. Annual income: categorised into quartiles, with the cut-off of £11 499 being the closest equivalent to the UK definition of poverty of 60% of median national household income: £40 000+, £17 500 to £39 999, £11 500 to £17 499, <£11 499, per annum.^41^
3. Educational level: university education, A-level and equivalent, GCSE/vocational, other/still studying, none.
4. Working status: full-time job versus no full-time job.

Smoking status in the STS was assessed by asking participants if they smoked cigarettes (daily or non-daily). High-risk (excessive) alcohol consumption was defined as a score of 8 or more (7 or more in women) on the full AUDIT.^42–44^

## Analysis

Data were analysed in R V.3.3.0 (R Development Core Team. R: a language and environment for statistical computing. R foundation for statistical computing, Vienna, Austria. 2008. http://www.R-project.org). The analysis plan was registered on the Open Science Framework prior to data analysis (https://osf.io/nbtwu/). Descriptive statistics were weighted for the STS and ATS using a rim (marginal) weighting technique. This involves an iterative sequence of weighting adjustments whereby separate nationally representative target profiles are set (for gender, working status, children in the household, age, social grade and region). This process is then repeated until all variables match the specified targets (for further details, see Fidler *et al*^36^ and Beard *et al*^35^). Missing data for income (42.3%), education (0.5%), employment status (0.3%), Government Office Region (0.2%) and smoking status (0.1%) were imputed using the multiple imputation package Amelia 11.^45^ Little's test suggested that income data may not have been missing at random.^46^ The number of imputed data sets was set to 20^47^ and results were combined using Rubin's rules.^46^

The prevalence of smoking and high-risk alcohol consumption was calculated overall and as a function of Government Office Region. Next, unadjusted weighted generalised linear models (with a log link and quasi-binomial distribution), using the Survey package,^48^ were run to assess whether associations existed between Government Office Region and the outcomes of interest. Results of these main regression models were tabulated and displayed graphically following the conversion of relative risk to per cent relative risk difference using the formulas: (_+ve_RR−1)×100 and (1−RR_−ve_)×100. Given the nominal nature of Government Office Region, the reference category in the analyses was chosen to reflect the region with the median prevalence, that is, the South West.^49^

Owing to high multicollinearity between SES measures, a composite score was derived using multiple correspondence analysis (MCA) applied using the FactoMineR package.^50^ Weights for the composite score comprised of those for the first three components.^51^ Since the composite score violated the assumption of linearity of the logit, it was categorised into quartiles: <7.8, <10.8, <14.1 and <19.8. Higher composite scores equal greater social disadvantage. Age also violated the assumption and was categorised into 16–24, 25–34, 35–44, 45–54, 55–64, 65+. STROBE guidelines were followed throughout.^52^

Additional unplanned sensitivity analyses were run to assess the moderating effects of sociodemographic characteristics on the association between Government Office Region with smoking and high-risk drinking. Moderation was assessed by including an interaction term in the regression models between Government Office Region and each sociodemographic characteristic, adjusting for all other sociodemographic characteristics. Results are given in terms of percentage relative risk difference compared with the reference category (ie, the South West).

## Results

### Main results

The prevalence of high-risk drinking was lowest in Greater London (9.0%), the East Midlands (10.3) and the West Midlands (8.7%), and highest in the North East (26.0%), North West (21.5%) and Yorkshire and the Humber (20.1%). There was also a clear North/South divide in smoking rates, with the highest prevalence in the North East (22.8%) and lowest prevalence in the South East (16.0%) (see table 2).

**Table 2:** Prevalence of smokers and high-risk drinkers by Government Office Region

Region	Total n	Smoker (n=7509)			High-risk drinkers (n=7869)		
		95% CI			95% CI		
		Per cent	Lower	Upper	Per cent	Lower	Upper
North East	2730	22.8	19.5	26.7	26.0	22.8	29.2
North West	7174	21.9	19.9	24.6	21.5	19.5	23.6
Yorkshire and the Humber	5495	21.5	19.1	25.0	20.1	17.7	22.5
East Midlands	4685	19.3	16.7	23.5	10.3	7.5	13.0
West Midlands	5483	17.8	15.4	19.9	8.7	6.2	11.2
East of England	6066	16.5	14.2	19.0	10.8	8.4	13.2
Greater London Authority	7994	16.4	14.4	19.0	9.0	6.9	11.1
South East	8748	16.0	14.1	17.9	15.7	13.8	17.7
South West	5547	18.7	16.3	21.0	14.5	12.1	17.0
Overall	53 922	18.5	17.8	19.3	14.6	13.8	15.4

**Figure 1:**
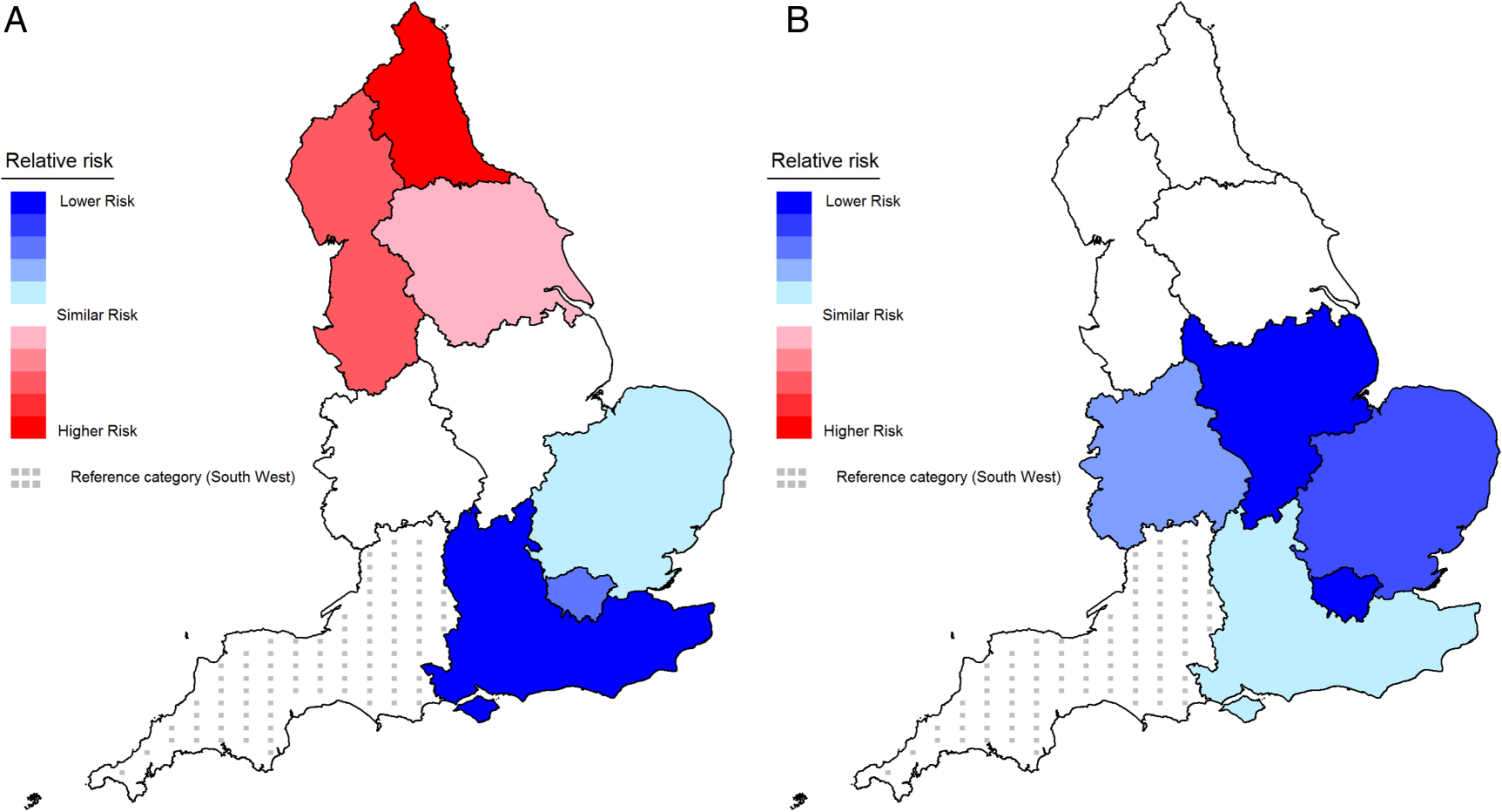
Association between Government Office Region and smoking: (A) unadjusted; (B) adjusted for gender, age, ethnicity and socioeconomic status. Note: this shows the relative risk difference for each region relative to the South West (dotted reference region). Increasing red tones reflect increasingly higher significant risk and increasing blue tones reflect increasingly lower significant risk. Regions shaded white have a similar risk to the South West. Online supplementary figure S9 labels the Government Office Regions in England.

Before adjustment, the regression analyses suggested that those in the North of England (North East, North West and Yorkshire and the Humber) had a greater risk of being a smoker compared with those in the South West, while those from the East of England, South East and Greater London Authority had a lower risk. No difference was found in the East and West Midlands (table 3 and figure 1A). After adjustment, the higher risk in the North of England dissipated; while the East Midlands, West Midlands, East of England, South East and Greater London showed a lower risk of smoking compared with the South West (table 3 and figure 1B).

**Table 3:** Association between Government Office Region with smoking and high-risk drinking status

	Smoker (n=7509)						High-risk drinkers (n=7869)					
	Unadjusted			Adjusted			Unadjusted			Adjusted		
	RR	95% CI		RR	95% CI		RR	95% CI		RR	95% CI	
		Lower	Upper		Lower	Upper		Lower	Upper		Lower	Upper
Government office region												
North East	1.22***	1.10	1.34	0.97	0.88	1.06	1.79***	1.62	1.99	1.67***	1.51	1.86
North West	1.17***	1.08	1.26	0.98	0.91	1.06	1.48***	1.36	1.62	1.42***	1.29	1.55
Yorkshire and the Humber	1.15***	1.06	1.24	1.03	0.95	1.11	1.38***	1.26	1.52	1.35***	1.22	1.48
East Midlands	1.03	0.94	1.13	0.86***	0.79	0.94	0.71***	0.63	0.80	0.69***	0.61	0.78
West Midlands	0.95	0.88	1.04	0.91*	0.84	0.99	0.60***	0.53	0.67	0.77***	0.69	0.87
East of England	0.88**	0.80	0.97	0.86***	0.79	0.94	0.74***	0.66	0.83	0.72***	0.64	0.81
Greater London Authority	0.87***	0.81	0.95	0.85***	0.79	0.92	0.62***	0.56	0.69	0.71***	0.64	0.79
South East	0.85***	0.78	0.93	0.92*	0.84	1.00	1.08	0.98	1.20	1.10	1.00	1.22
South West (reference)												
Gender												
Male (reference)												
Female				0.86	0.83	0.89				0.64***	0.62	0.68
Age												
16–24 (reference)												
25–34				1.20***	1.13	1.27				0.68***	0.64	0.73
35–44				1.09**	1.03	1.16				0.62***	0.58	0.67
45–54				0.95	0.89	1.00				0.61***	0.57	0.66
55–64				0.74***	0.69	0.78				0.49***	0.46	0.53
65+				0.34***	0.32	0.36				0.24***	0.22	0.26
Ethnicity												
White (reference)												
Mixed/multiple ethnic group				0.96	0.83	1.11				0.87	0.73	1.04
Asian or British Asian				0.41***	0.38	0.45				0.08***	0.07	0.11
Black African/Caribbean/Black British				0.40***	0.35	0.46				0.25***	0.20	0.32
Other ethnic group				0.80**	0.69	0.92				0.42***	0.31	0.57
Composite SES score												
1st quartile (high) (reference)												
2nd quartile				1.57***	1.45	1.69				0.95	0.89	1.02
3rd quartile				2.16***	2.01	2.32				0.86***	0.81	0.92
4th quartile (low)				3.13***	2.92	3.35				0.69***	0.64	0.75

***Significant at p<0.001; **significant at p<0.01; *significant at p<0.05.

10.1136/bmjopen-2016-014210.supp1 supplementary figures

**Figure 2:**
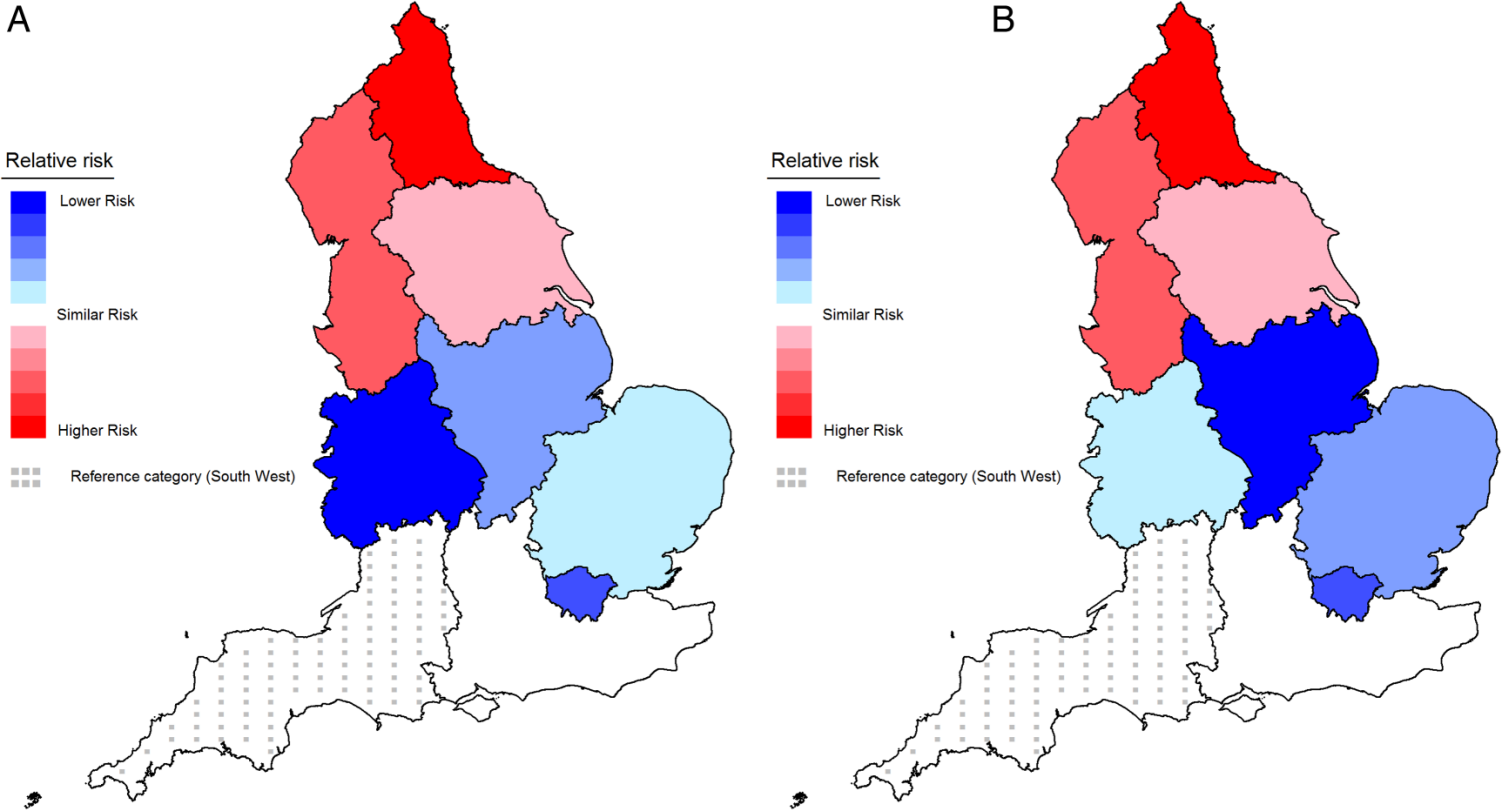
Association between Government Office Region and high-risk drinking: (A) unadjusted; (B) adjusted for gender, age, ethnicity and socioeconomic status (reference region: South West). Note: this shows the relative risk difference for each region relative to the South West (dotted reference region). Increasing red tones reflect increasingly higher significant risk and increasing blue tones reflect increasingly lower significant risk. Regions shaded white have a similar risk to the South West. Online supplementary figure S9 labels the Government Office Regions in England.

Before adjustment, those in the North of England had a greater risk of reporting being a high-risk drinker, while those in all other regions, except the South East of England, had a lower risk compared with the South West (table 3 and figure 2A). After adjustment, a similar pattern was found (table 3 and figure 2B).

### Supplementary analyses

Online supplementary figures S1–S8 show the moderation effects of sociodemographic characteristics on the association between Government Office Region with smoking and high-risk drinking. Significant interaction effects were found for all sociodemographic characteristics. Lower prevalence of smoking in the West Midlands and Greater London Authority compared with the South West was the only event among men (p<0.05), while the relative risk difference of excessive alcohol consumption was significantly larger among men in the North West than women (p<0.05). There appeared to be a trend towards increasing risk of smoking and high-risk drinking with age in the North of England compared with the reference region (p<0.05), with relative risk differences being highest for smoking among those aged 55+ years of age. In the East Midlands and Greater London Authority, smoking and alcohol consumption rates were only significantly lower than the South West among 16–24-year-olds, with older age groups having a significantly higher risk of both behaviours (p<0.05). In the South East, the prevalence of high-risk drinking relative to the South West increased exponentially with age.

Whereas those of higher SES had a greater risk of smoking in the North West and a similar risk of smoking in Greater London Authority relative to the South West, those of lower SES in these regions had a significantly lower risk of smoking (p<0.05). There was also a significant trend towards an increased likelihood of high-risk drinking in Greater London Authority with increasing social grade (p<0.05). In general, those of white ethnicity appeared to have a similar risk of smoking across regions, a higher risk of high-risk drinking in Northern regions, and a lower risk of high-risk drinking in Southern regions, compared with the South West (p<0.05). Risk of both behaviours was lower among most ethnic minority groups (p<0.05).

## Discussion

This study assessed the association between Government Office Region with smoking and alcohol in a large population survey in England. In unadjusted analyses, there was a clear North–South divide in smoking, while high-risk alcohol consumption was common in the North and less common in ‘central England’. The regional differences in the North for smoking but not high-risk drinking appeared to be explained somewhat by sociodemographic disparities. After adjustment, the lower rates of smoking and high-risk drinking in the East of England and Greater London Authority remained. Moderation analyses indicated that the higher risk of smoking in the North West compared with the South West was largely driven by those of an older age and higher SES, while higher prevalence of excessive drinking in Northern regions was driven by older men of white ethnicity. The lower risk of both behaviours in Southern regions and the Midlands appeared to stem from ethnic minority groups and those of a younger age. In London, the lower prevalence relative to the South West also appeared to be driven partly by men of a lower SES. The finding in the unadjusted analyses of a North–South divide in smoking is largely consistent with the findings from other surveys. For example, the Integrated Household Survey in 2014^10^ noted a prevalence of 17.3% in London, 17.2% in the South East and 17.3% in the South West. This was significantly lower than the 22.3% in the North East, 20.1% in the North West and 20.3% in Yorkshire and The Humber. Previous population surveys have also similarly established a higher prevalence of high-risk drinking in northern regions and lower prevalence in ‘central England’. The Adult Psychiatric Morbidity Survey in 2016 reported that the proportion of individuals drinking excessively (AUDIT score >8) was between 22% and 25% in Northern regions, dropping to 18% in the East Midlands and 19% in the West Midlands.^53^ The findings are less consistent for the South of England, with sales data and the Health Survey for England finding a higher than average consumption rate in the South West,^13^ ^30^ while other surveys report consumption estimates for this region which are about the same, or slightly lower than the national average.^12^ ^54^ Although it is difficult to attribute these discrepancies to any single factor, tourism and differences in survey design may play a role.^13^

This study builds on previous findings by suggesting that some of the regional variations in smoking, particularly the presence of a North/South divide, can be explained by sociodemographic characteristics. In contrast, variations in high-risk drinking could not be wholly attributed to sociodemographic differences between regions. This suggests a need to identify other contributing factors. One possible factor is local-level differences in policies and disparities in the distribution of public healthcare facilities. For example, since the move of commissioning of Stop Smoking Services and Alcohol Services to local authorities in 2013, a fragmented system of support exists across England.^55^ The divide in England could also be a residual effect of the historically greater use of tobacco in the North during the early 1900s due to the dominance of tobacco manufacturing in these regions.^56^ Social and psychological factors offer another source of variation, and include cultural differences, attitudes and beliefs towards drinking, levels of integration in communities and political orientation.^57^ ^58^ Environmental factors should also be considered, such as loss of infrastructure and urban decline in northern England.^59^

The moderation analysis indicates whether particular sociodemographic characteristics were differentially associated with the behaviours across the different regions. Higher prevalence of smoking and high-risk drinking in Northern regions was most evident in older white men of a higher SES compared with their counterparts in the South West, while the lower prevalence in the South and Midlands was most evident among younger ethnic minority groups compared with their counterparts in other regions of the country. These within sociodemographic category differential risks across regions are most likely explained in part by the factors noted above, including variations in beliefs, views and cultural attitudes towards smoking and drinking.^60^ ^61^ For example, men residing in Northern regions are more likely to support the traditional ‘strong masculine breadwinner’ stereotype, which is associated with increased use of tobacco products,^58^ ^62^ while differences within ethnic minority subgroups may reflect levels of integration and whether they are first, second or third-generation immigrants.^63^ An additional finding of interest was the non-linear monotonic associations between the two health behaviours and the sociodemographic characteristics. For example, the relative risk difference in Greater London Authority of smoking decreased exponentially with SES and peaked among those aged 55–64 before declining again. This is consistent with previous studies suggesting that alcohol-related and smoking-related harm are concentrated disproportionately in the most socially deprived.^11^ ^27^

These findings have several implications for policymakers and researchers. They suggest that high-risk drinking spans the spectrum of sociodemographic groups. This may reflect in part the Alcohol Harm Paradox, with higher consumption rates among the more affluent in society but greater experienced harm among the most socially disadvantaged.^14^ In contrast, the result that regional differences in smoking appear to be driven by sociodemographic variation implies that tackling the most at-risk subgroups across the country may reduce variation in smoking rates. Policies of allocating a greater proportion of health resources to poorer areas have been shown to be associated with declining inequalities in mortality,^64^ while decreases in unemployment in deprived areas prior to the economic crisis in 2008 were associated with reductions in inequalities in male life expectancy between areas.^65^

The capacity of the UK government to address regional imbalances has been limited somewhat by the dismantling of regional administrative structures in recent years, including Government Offices, Regional Development Agencies and Strategic Health Authorities.^60^ This has resulted in a fragmented system of support for smokers and dependent drinkers. Although Stop Smoking Services are one of the most cost-effective life preserving services offered by local authorities, and local-level intensive alcohol licensing policies (known as Cumulative Impact Zones) have been shown to result in declines in rates of violent crimes, sexual crimes and public order offences, substantial variation exists across England in terms of their effectiveness and scale of implementation.^55^ ^66–68^

Regional variations are also likely to be exacerbated over the coming years if radical changes in strategy do not occur. The current economic strategy of the Government has focused on Greater London Authority and the South West, despite the 2008 economic crisis affecting the North of England disproportionately,^69^ and has resulted in a number of reforms to the welfare system which are increasing the economic divide.^34^

This study benefits from the use of a representative survey about smoking and drinking conducted on a large sample of the adult population in England. To the best of our knowledge, this paper provides the most up-to-date information on regional differences in smoking and high-risk drinking accounting for disparities in gender, SES, ethnicity and age. A strength of this study is the use of AUDIT to categorise high-risk drinkers, as it incorporates consumption, harm and possible dependence measures. However, as with other population-based studies, a number of limitations need to be considered. While the sample was designed to be representative, there is a risk of bias in terms of the characteristics of those who agree to participate. There is also a risk that respondents may underestimate or fail to report their drinking and smoking. Large amounts of data were missing for income, which may have affected the quality of adjustment for SES, although we have shown previously that derived composite measures have good reliability due to their recognition of the multifaceted nature of SES.^27^ There are also limitations with the use of AUDIT, including the need to retrospectively recall average intake in the past and self-reported harm in the form of injuries and other concerns. Nonetheless, a strong association has been found between self-reported high-risk drinking using the AUDIT and alcohol-related illness, social problems, hospital admission and mortality.^70^ This paper also only considered patterns of smoking and alcohol use at the Government Office Region level, an approach taken by other population surveys (eg, the Health Survey for England and Integrated Household Survey) due to the historical link until April 2013 with strategic health authorities. This ensured enough power to be able to assess the impact of sociodemographic characteristics on regional variations. Since April 2013, commissioning of stop smoking and alcohol services has been moved to local authority control. Thus, future studies may wish to consider variations as this more micro-geographical level.^71^ Additionally, this paper was only interested in the prevalence of smoking and high-risk drinking. It would be of interest to consider regional variations in key-related behaviours such as the number of cigarettes smoked per day, binge drinking and attempts to quit or cut down on smoking and alcohol intake. For example, previous studies suggest that regional variations differ according to the measure of alcohol consumption used (eg, volume vs frequency).^31^ Finally, although these findings relate to England specifically, which has the largest difference in economic output between regions of any country in Europe, it is most likely that comparable variation would be found in other countries.^34^

In conclusion, smoking and high-risk drinking appear to be less common in ‘central England’ than in the rest of the country. Regional differences in smoking, but not those in high-risk drinking, are explained to some extent by sociodemographic disparities. These findings have a number of implications for policymakers and researchers, including the need to determine what other factors may account for regional variations in high-risk drinking.

## References

[1] Brown J, West R, Angus C Comparison of brief interventions in primary care on smoking and excessive alcohol consumption: a population survey in England. Br J Gen Pract 2016;66:e1–9. 10.3399/bjgp16X683149 26719481 PMC4684029

[2] Ellison J Reducing harmful drinking. 2013 https://www.gov.uk/government/policies/reducing-harmful-drinking

[3] Lee PN, Forey BA, Coombs KJ Systematic review with meta-analysis of the epidemiological evidence in the 1900s relating smoking to lung cancer. BMC Cancer 2012;12:385 10.1186/1471-2407-12-385 22943444 PMC3505152

[4] Connor J Alcohol consumption as a cause of cancer. Addiction 2017;112:222–8. 10.1111/add.13477 27442501

[5] Office for National Statistics. Alcohol Related Deaths in the United Kingdom: Registered in 2014. 2016 https://www.ons.gov.uk/peoplepopulationandcommunity/healthandsocialcare/causesofdeath/bulletins/alcoholrelateddeathsintheunitedkingdom/registeredin2014

[6] Health and Social Care Information Centre. Statistics on NHS Stop Smoking Services in England—April 2014 to March 2015. 2015 http://www.hscic.gov.uk/catalogue/PUB18002

[7] Fone DL, Farewell DM, White J Socioeconomic patterning of excess alcohol consumption and binge drinking: a cross-sectional study of multilevel associations with neighbourhood deprivation. BMJ Open 2013;3:pii: e002337 10.1136/bmjopen-2012-002337 PMC3641461 23587771

[8] Siegler V, Al-Hamad A, Johnson B Social inequalities in alcohol-related adult mortality by National Statistics Socio-economic Classification, England and Wales, 2001–03. 2011. http://www.ons.gov.uk/ons/rel/hsq/health-statistics-quarterly/no--50--summer-2011/social-inequalities-in-alcohol-related-adult-mortality-by-national-statistics-socio-economic-classification--england-and-wales--2001-03.pdf 10.1057/hsq.2011.7 21647087

[9] Law MR, Morris JK Why is mortality higher in poorer areas and in more northern areas of England and Wales? J Epidemiol Community Health 1998;52:344–52. 10.1136/jech.52.6.344 9764254 PMC1756726

[10] Health & Social Care Information Centre. Statistics on Smoking, England—2015. 2015 http://digital.nhs.uk/catalogue/PUB17526/stat-smok-eng-2015-rep.pdf

[11] Office for National Statistics. Do smoking rates vary between more and less advantaged areas? 2014 http://webarchive.nationalarchives.gov.uk/20160105160709/http://www.ons.gov.uk/ons/rel/disability-and-health-measurement/do-smoking-rates-vary-between-more-and-less-advantaged-areas-/2012/sty-smoking-rates.html

[12] Twigg L, Moon G The spatial and temporal development of binge drinking in England 2001–2009: an observational study. Soc Sci Med 2013;91:162–7. 10.1016/j.socscimed.2013.03.023 23608600

[13] Robinson M, Shipton D, Walsh D Regional alcohol consumption and alcohol-related mortality in Great Britain: novel insights using retail sales data. BMC Public Health 2015;15:1–9. 10.1186/1471-2458-15-1 25563658 PMC4324675

[14] Alcohol Research UK. Alcohol Research UK Final Report Understanding the alcohol harm paradox in order to focus the development of interventions. 2015 http://www.cph.org.uk/wp-content/uploads/2015/03/alcohol-harm-paradox-final-report.pdf

[15] Office for National Statistics. Alcohol Related Deaths in the United Kingdom: Registered in 2014. 2014 https://www.ons.gov.uk/peoplepopulationandcommunity/healthandsocialcare/causesofdeath/bulletins/alcoholrelateddeathsintheunitedkingdom/registeredin2014

[16] Breakwell C, Baker A, Griffiths C Trends and geographical variations in alcohol-related deaths in the United Kingdom, 1991–2004. Health Stat Q 2007;33:6–24. 17373379

[17] Erskine S, Maheswaran R, Pearson T Socioeconomic deprivation, urban–rural location and alcohol-related mortality in England and Wales. BMC Public Health 2010;10:99 10.1186/1471-2458-10-99 20184763 PMC2841677

[18] Marmot M, Friel S, Bell R Closing the gap in a generation: health equity through action on the social determinants of health. Lancet 2008;372:1661–9. 10.1016/S0140-6736(08)61690-6 18994664

[19] Department of Health. Tackling health inequalities: 2006–8 data and policy update for the 2010 national target. http://www.dh.gov.uk/dr_consum_dh/groups/dh_digitalassets/@dh/@en/@ps/@sta/@perf/documents/digitalasset/dh_109468.pdf 2009.

[20] Hacking JM, Muller S, Buchan IE Trends in mortality from 1965 to 2008 across the English north–south divide: comparative observational study. BMJ 2011;342:d508 10.1136/bmj.d508 21325004 PMC3039695

[21] Martin R Remapping British regional policy: the end of the North–South divide? Regional Studies 1993;27:797–805.

[22] Office for National Statistics. Overview of the UK population: February 2016. 2016 https://www.ons.gov.uk/peoplepopulationandcommunity/populationandmigration/populationestimates/articles/overviewoftheukpopulation/february2016

[23] Institute of Alcohol Studies. Alcohol, Health inequalities and the harm paradox: why some groups face greater problems despite consuming less alcohol. 2014. http://www.ias.org.uk/uploads/pdf/IAS%20reports/IAS%20report%20Alcohol%20and%20health%20inequalities%20FULL.pdf

[24] Office for National Statistics. South West had the oldest population in the UK in 2012. 2013 http://webarchive.nationalarchives.gov.uk/20160105160709/http://www.ons.gov.uk/ons/rel/regional-trends/region-and-country-profiles/region-and-country-profiles---key-statistics-and-profiles--october-2013/key-statistics-and-profiles---south-west--october-2013.html

[25] ESRC Centre on Dynamics of Ethnicity (CoDE). How has ethnic diversity grown 1991–2001–2011? 2012 http://www.ethnicity.ac.uk/medialibrary/briefings/dynamicsofdiversity/how-has-ethnic-diversity-grown-1991-2001-2011.pdf

[26] Britton A, Ben-Shlomo Y, Benzeval M Life course trajectories of alcohol consumption in the United Kingdom using longitudinal data from nine cohort studies. BMC Med 2015;13:47 10.1186/s12916-015-0273-z 25858476 PMC4351673

[27] Beard E, Brown J, West R Deconstructing the alcohol harm paradox: a population based survey of adults in England. PLoS ONE 2016;11:e0160666 10.1371/journal.pone.0160666 27682619 PMC5040414

[28] Hiscock R, Bauld L, Amos A Socioeconomic status and smoking: a review. Ann N Y Acad Sci 2012;1248:107–23. 10.1111/j.1749-6632.2011.06202.x 22092035

[29] Hiscock R, Bauld L, Amos A Smoking and socioeconomic status in England: the rise of the never smoker and the disadvantaged smoker. J Public Health (Oxf) 2012;34:390–6. 10.1093/pubmed/fds012 22375070 PMC3425348

[30] Health & Social Care Information Centre. National Statistics Health Survey for England, 2014: Trend tables [NS]. 2015 http://www.hscic.gov.uk/catalogue/PUB19297

[31] Castillo JM, Jivraj S, Ng Fat L The regional geography of alcohol consumption in England: comparing drinking frequency and binge drinking. Health Place 2017;43:33–40. 10.1016/j.healthplace.2016.11.007 27894017

[32] Duncan C, Jones K, Moon G Smoking and deprivation: are there neighbourhood effects? Soc Sci Med 1999;48:497–505. 10.1016/S0277-9536(98)00360-8 10075175

[33] Möller H, Haigh F, Harwood C Rising unemployment and increasing spatial health inequalities in England: further extension of the North–South divide. J Public Health (Oxf) 2013;35:313–21. 10.1093/pubmed/fds085 23292091

[34] Whitehead M, McInroy N, Bambra C Due North: report of the inquiry on health equity for the North. Liverpool: University of Liverpool and the Centre for Economic Strategies, 2014.

[35] Beard E, Brown J, West R Protocol for a national monthly survey of alcohol use in England with 6-month follow-up: ‘The Alcohol Toolkit Study’. BMC Public Health 2015;15:230 10.1186/s12889-015-1542-7 25884652 PMC4363185

[36] Fidler JA, Shahab L, West O ‘The smoking toolkit study’: a national study of smoking and smoking cessation in England. BMC Public Health 2011;11:479 10.1186/1471-2458-11-479 21682915 PMC3145589

[37] Allen JP, Litten RZ, Fertig JB A review of research on the Alcohol Use Disorders Identification Test (AUDIT). Alcohol Clin Exp Res 1997;21:613–9. 10.1111/j.1530-0277.1997.tb03811.x 9194913

[38] Babor TF, Higgins-Biddle JC, Saunders JB AUDIT: the alcohol use disorders identification test: guidelines for use in primary care. 2nd edn Geneva, Switzerland: WHO, 2001.

[39] Office for National Statistics. Ethnicity and National Identity in England and Wales: 2011. 2012 https://www.ons.gov.uk/peoplepopulationandcommunity/culturalidentity/ethnicity/articles/ethnicityandnationalidentityinenglandandwales/2012-12-11

[40] Collis D Social grade: A classification tool—Bite sized through piece. 2009 https://www.ipsos-mori.com/DownloadPublication/1285_MediaCT_thoughtpiece_Social_Grade_July09_V3_WEB.pdf

[41] Pensions DfWa. Households below average income. 2015 https://www.gov.uk/government/uploads/system/uploads/attachment_data/file/437246/households-below-average-income-1994-95-to-2013-14.pdf

[42] Casswell S, Meier P, MacKintosh AM The International Alcohol Control (IAC) study—evaluating the impact of alcohol policies. Alcohol Clin Exp Res 2012;36:1462–7. 10.1111/j.1530-0277.2012.01738.x 22404733

[43] Caviness CM, Hatgis C, Anderson BJ Three brief alcohol screens for detecting hazardous drinking in incarcerated women. J Stud Alcohol Drugs 2009;70:50–4. 10.15288/jsad.2009.70.50 19118391 PMC2629634

[44] National Institute on Alcohol Abuse and Alcoholism. Helping patients who drink too much: a clinician's guide. Bethesda, MD: National Institute on Alcohol Abuse and Alcoholism, 2007.

[45] Honaker J, King G, Blackwell M Amelia II: a program for missing data. J Stat Softw 2011;45:1–47. 10.18637/jss.v045.i07

[46] Little RJ, Rubin DB The analysis of social science data with missing values. Sociol Method Res 1989;18:292–326. 10.1177/0049124189018002004

[47] Graham JW, Olchowski AE, Gilreath TD How many imputations are really needed? Some practical clarifications of multiple imputation theory. Prev Sci 2007;8:206–13. 17549635 10.1007/s11121-007-0070-9

[48] Lumley T Analysis of complex survey samples. Journal of Statistical Software 2004;9:1–19.

[49] Batista-Foguet J, Fortiana J, Currie C Socio-economic indexes in surveys for comparisons between countries. Soc Indic Res 2004;67:315–32. 10.1023/B:SOCI.0000032341.14612.b8

[50] Lê S, Josse J, & Husson F FactoMineR: An R Package for Multivariate Analysis. Journal of Statistical Software 2008;25:1–18.

[51] Ram R Composite indices of physical quality of life, basic needs fulfilment, and income: a ‘principal component’ representation. J Dev Econ 1982;11:227–47. 10.1016/0304-3878(82)90005-0

[52] Von Elm E, Altman DG, Egger M The Strengthening the Reporting of Observational Studies in Epidemiology (STROBE) statement: guidelines for reporting observational studies. Prev Med 2007;45:247–51. 10.1016/j.ypmed.2007.08.012 17950122

[53] NHS Digital. Adult Psychiatric Morbidity Survey: Survey of Mental Health and Wellbeing, England, 2014 [NS]. https://www.gov.uk/government/statistics/adult-psychiatric-morbidity-survey-mental-health-and-wellbeing-england-2014 2016.

[54] Deacon L Indications of public health in the English regions: alcohol. Association of Public Health Observatories, 2007.

[55] West R, May S, West M Performance of English stop smoking services in first 10 years: analysis of service monitoring data. BMJ 2013;347:f4921. 23963106 10.1136/bmj.f4921

[56] Goodman J Tobacco in history: the cultures of dependence. Routledge, 2005.

[57] Measham F, Brain K ‘Binge’ drinking, British alcohol policy and the new culture of intoxication. Crime Media Cult 2005;1:262–83. 10.1177/1741659005057641

[58] Duncan S, Smith D Family geographies and gender cultures. Soc Pol Soc 2002;1:21–34. 10.1017/S1474746402001045

[59] González S The North/South divide in Italy and England: discursive construction of regional inequality. European Urban and Regional Studies, 2010.

[60] Bambra C, Barr B, Milne E North and South: addressing the English health divide. J Public Health (Oxf) 2014;36:183–6. 10.1093/pubmed/fdu029 24860153

[61] Blackaby DH, Manning DN The north–south divide: questions of existence and stability. Econ J 1990;100:510–27. 10.2307/2234137

[62] Hunt K, Hannah MK, West P Contextualizing smoking: masculinity, femininity and class differences in smoking in men and women from three generations in the west of Scotland. Health Educ Res 2004;19:239–49 10.1093/her/cyg061 15140844

[63] Reiss K, Sauzet O, Breckenkamp J How immigrants adapt their smoking behaviour: comparative analysis among Turkish immigrants in Germany and the Netherlands. BMC Public Health 2014;14:844 10.1186/1471-2458-14-844 25124365 PMC4150979

[64] Barr B, Bambra C, Whitehead M The impact of NHS resource allocation policy on health inequalities in England 2001–11: longitudinal ecological study 2014;348:g3231. 10.1136/bmj.g3231 PMC4035504 24865459

[65] Barr B, Taylor-Robinson D, Whitehead M Impact on health inequalities of rising prosperity in England 1998-2007, and implications for performance incentives: longitudinal ecological study. BMJ 2012;345:e7831 10.1136/bmj.e7831 23212879 PMC3514473

[66] De Vocht F, Heron J, Campbell R Testing the impact of local alcohol licencing policies on reported crime rates in England. J Epidemiol Community Health 2017;71:137–45. 10.1136/jech-2016-207753 27514936 PMC5284476

[67] Egan M, Brennan A, Buykx P Local policies to tackle a national problem: comparative qualitative case studies of an English local authority alcohol availability intervention. Health Place 2016;41:11–8. 10.1016/j.healthplace.2016.06.007 27419612

[68] Brose LS, West R, McDermott MS What makes for an effective stop-smoking service? Thorax 2011;66:924–6. 10.1136/thoraxjnl-2011-200251 21709164

[69] Adonis A Mending the fractured economy: smarter state, better jobs. 2014 http://www.policyforum.labour.org.uk/uploads/editor/files/Adonis_Review.pdf

[70] Conigrave KM, Saunders JB, Reznik RB Predictive capacity of the AUDIT questionnaire for alcohol-related harm. Addiction 1995;90:1479–85. 10.1111/j.1360-0443.1995.tb02810.x 8528033

[71] ONS. Inequality in Health and Life Expectancies within Upper Tier Local Authorities: 2009 to 2013 2015 http://www.ons.gov.uk/peoplepopulationandcommunity/healthandsocialcare/healthandlifeexpectancies/bulletins/inequalityinhealthandlifeexpectancieswithinuppertierlocalauthorities/2009to2013

