# Supplementary material for: Healthier central England or North–South divide? Analysis of national survey data on smoking and high-risk drinking
Source: BMJ Open. 2017 Mar 1;7(3):e014210. doi: 10.1136/bmjopen-2016-014210 (PMC5353327; doi:10.1136/bmjopen-2016-014210)
Supplement: supplementary figures [file bmjopen-2016-014210supp_figures.pdf]

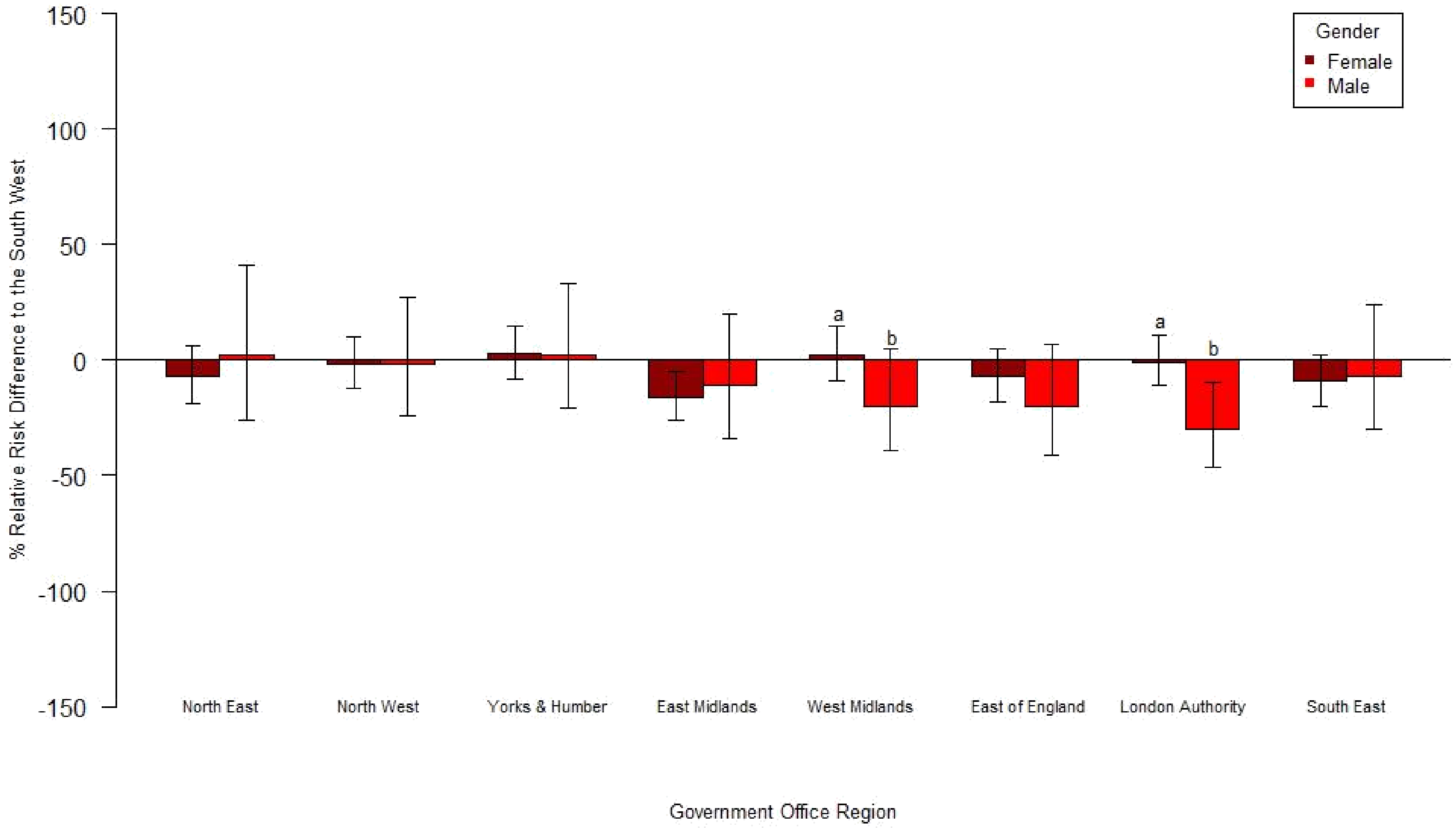

Supplementary Figure 1: Moderation effect of gender on the association between Government Office Region and smoking

Note: Bars reflect 95% confidence intervals; Confidence intervals which do not straddle 0 indicate a significantly different risk of smoking in that region relative to the South West for the subgroup under investigation; 'a' and 'b' reflect a significant interaction effect at p<0.05 i.e. the relative risk difference of smoking for sub-group 'a' is significantly different to subgroup 'b'

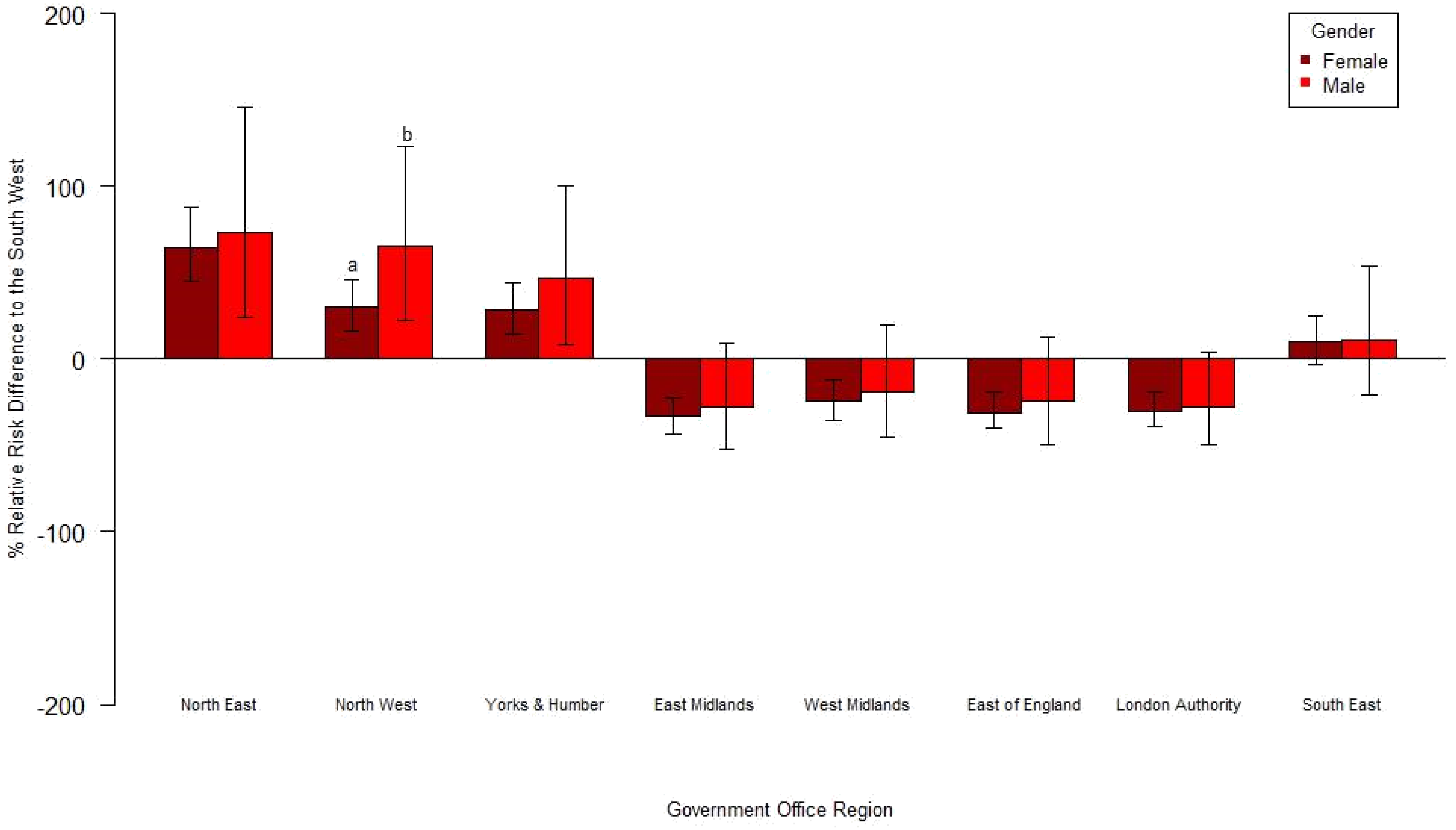

Supplementary Figure 2: Moderation effect of gender on the association between Government Office Region and high-risk drinking

Note: Bars reflect 95% confidence intervals; Confidence intervals which do not straddle 0 indicate a significantly different risk of high-risk drinking in that region relative to the South West for the subgroup under investigation; 'a' and 'b' reflect a significant interaction effect at p<0.05 i.e. the relative risk difference of high-risk drinking for sub-group 'a' is significantly different to subgroup 'b'

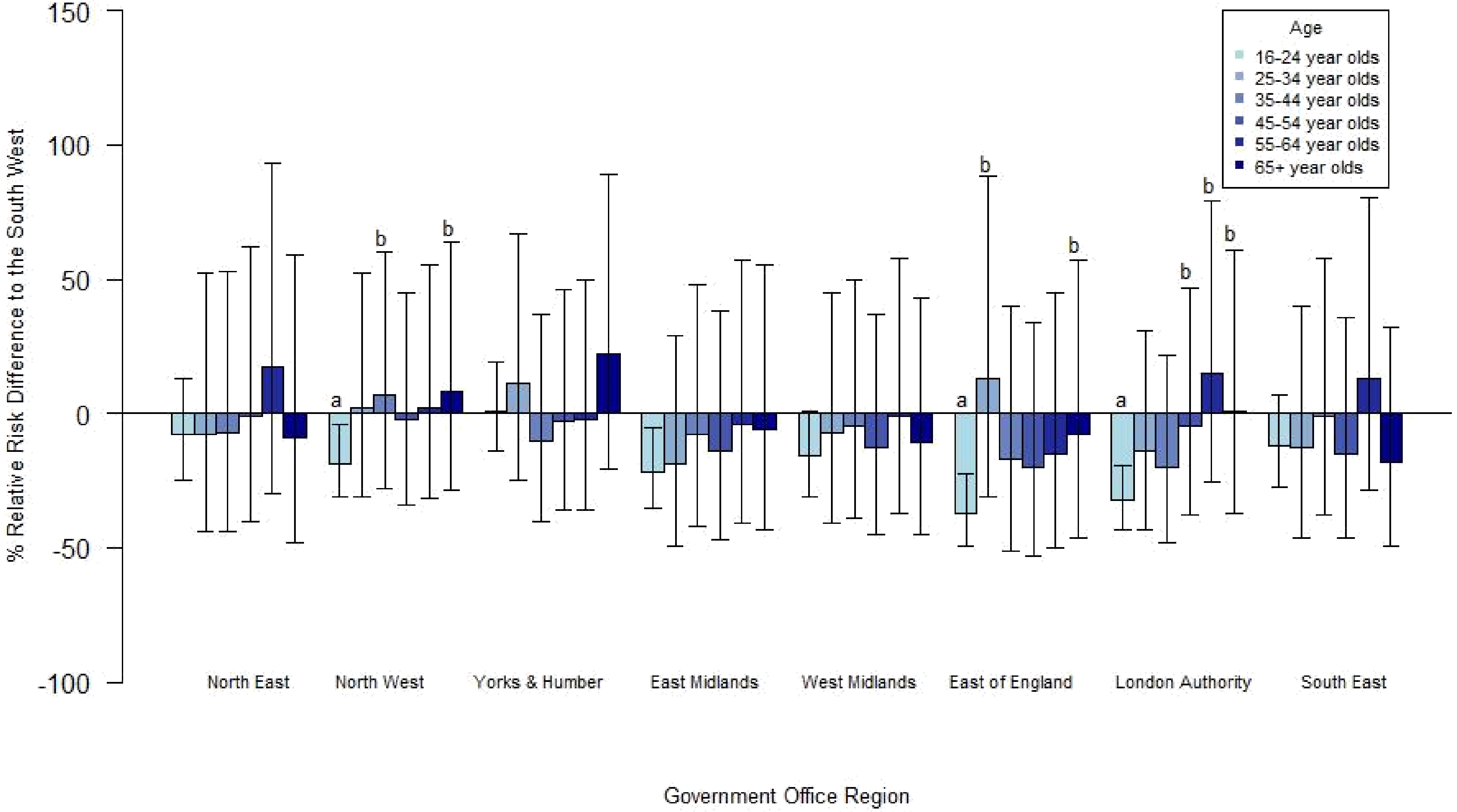

Supplementary Figure 3: Moderation effect of age on the association between Government Office Region and smoking

Note: Bars reflect 95% confidence intervals; Confidence intervals which do not straddle 0 indicate a significantly different risk of smoking in that region relative to the South West for the subgroup under investigation; 'a' and 'b' reflect a significant interaction effect at p<0.05 i.e. the relative risk difference of smoking for sub-group 'a' is significantly different to subgroup 'b'

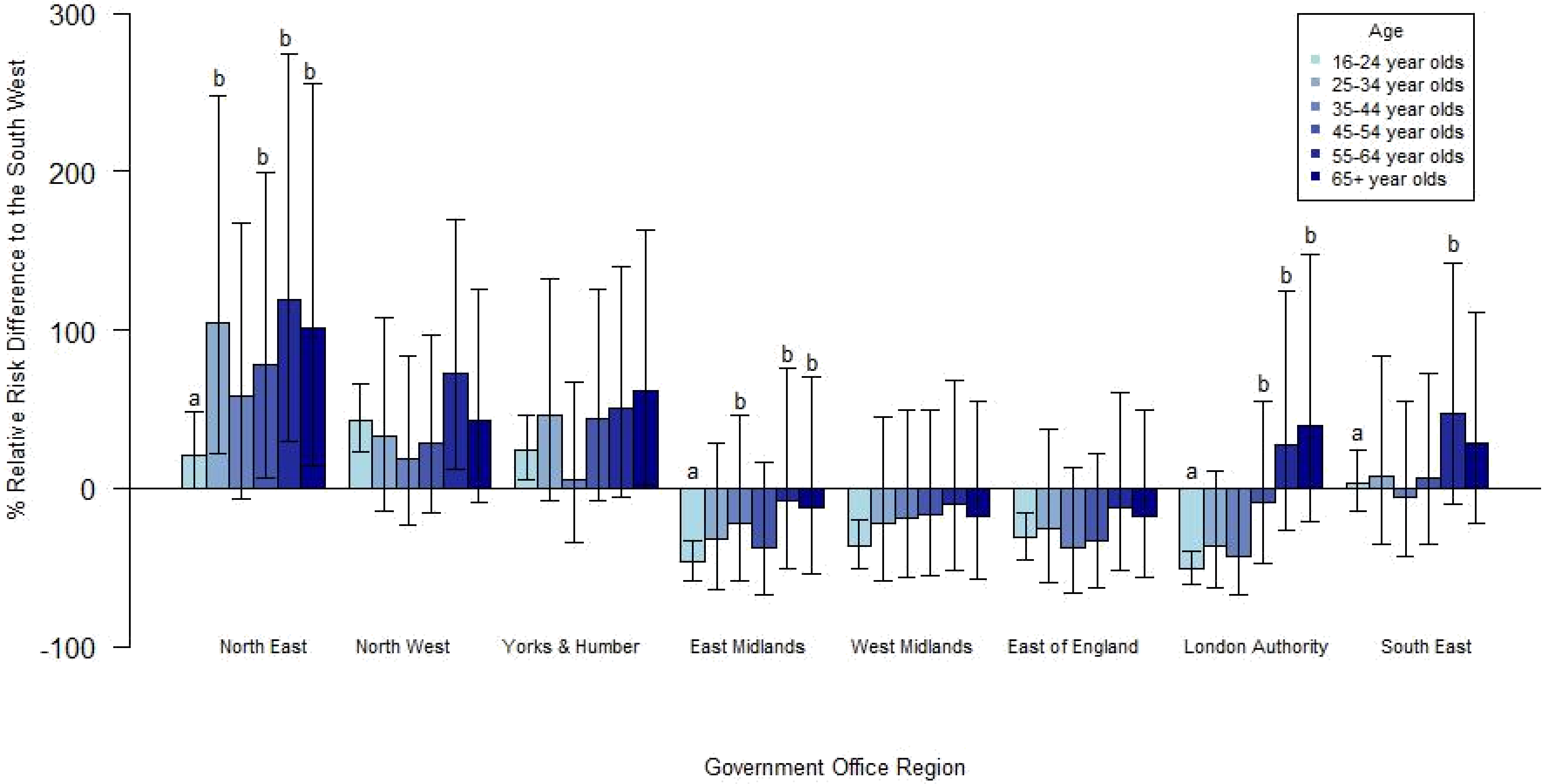

Supplementary Figure 4: Moderation effect of age on the association between Government Office Region and high-risk drinking

Note: Bars reflect 95% confidence intervals; Confidence intervals which do not straddle 0 indicate a significantly different risk of high-risk drinking in that region relative to the South West for the subgroup under investigation; 'a' and 'b' reflect a significant interaction effect at p<0.05 i.e. the relative risk difference of high-risk drinking for sub-group 'a' is significantly different to subgroup 'b'

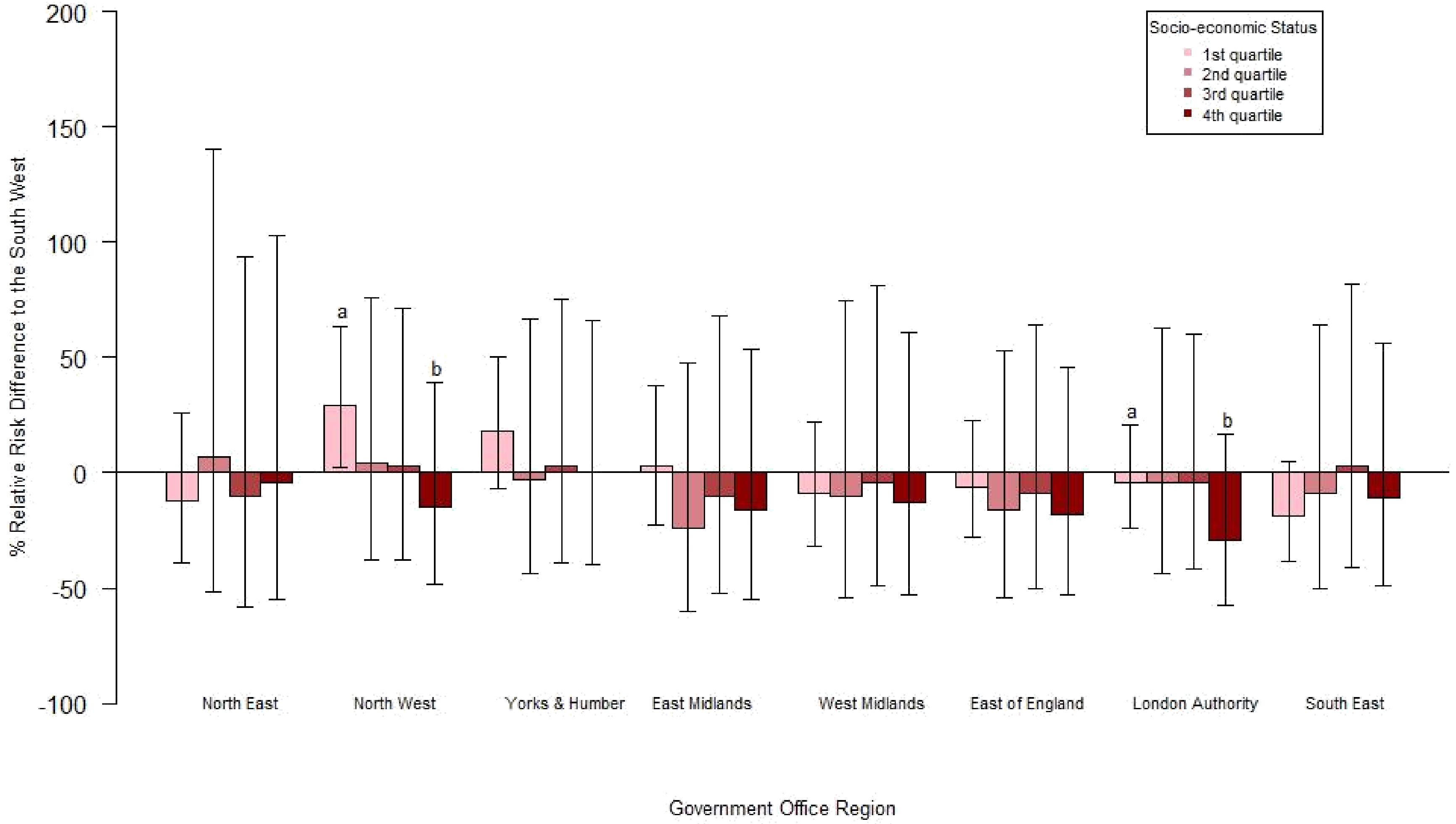

Supplementary Figure 5: Moderation effect of socio-economic status on the association between Government Office Region and smoking

Note: Bars reflect 95% confidence intervals; Confidence intervals which do not straddle 0 indicate a significantly different risk of smoking in that region relative to the South West for the subgroup under investigation; 'a' and 'b' reflect a significant interaction effect at p<0.05 i.e. the relative risk difference of smoking for sub-group 'a' is significantly different to subgroup 'b'

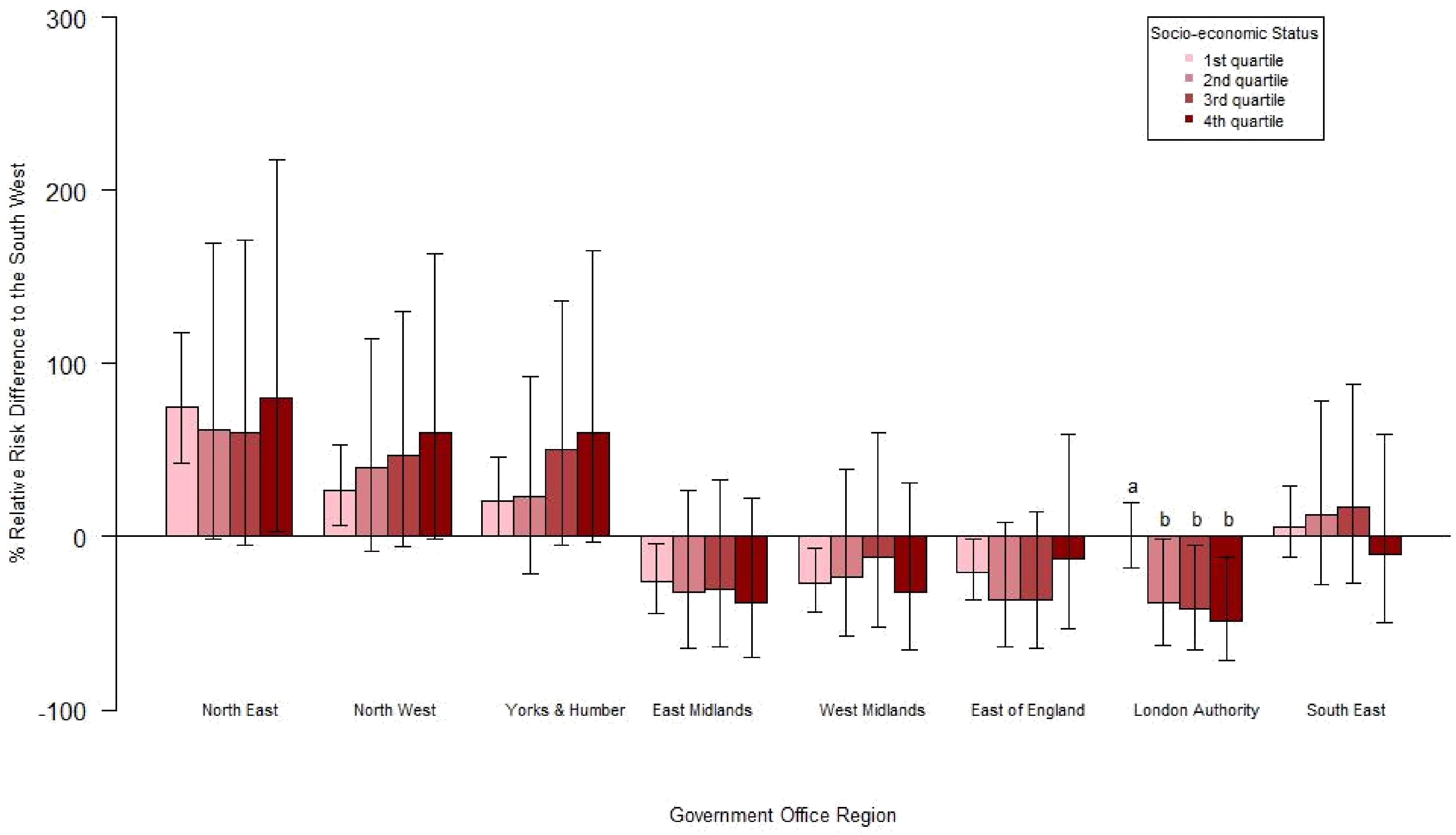

Supplementary Figure 6: Moderation effect of socio-economic status on the association between Government Office Region and high-risk drinking

Note: Bars reflect 95% confidence intervals; Confidence intervals which do not straddle 0 indicate a significantly different risk of high-risk drinking in that region relative to the South West for the subgroup under investigation; 'a' and 'b' reflect a significant interaction effect at p<0.05 i.e. the relative risk difference of high-risk drinking for sub-group 'a' is significantly different to subgroup 'b'

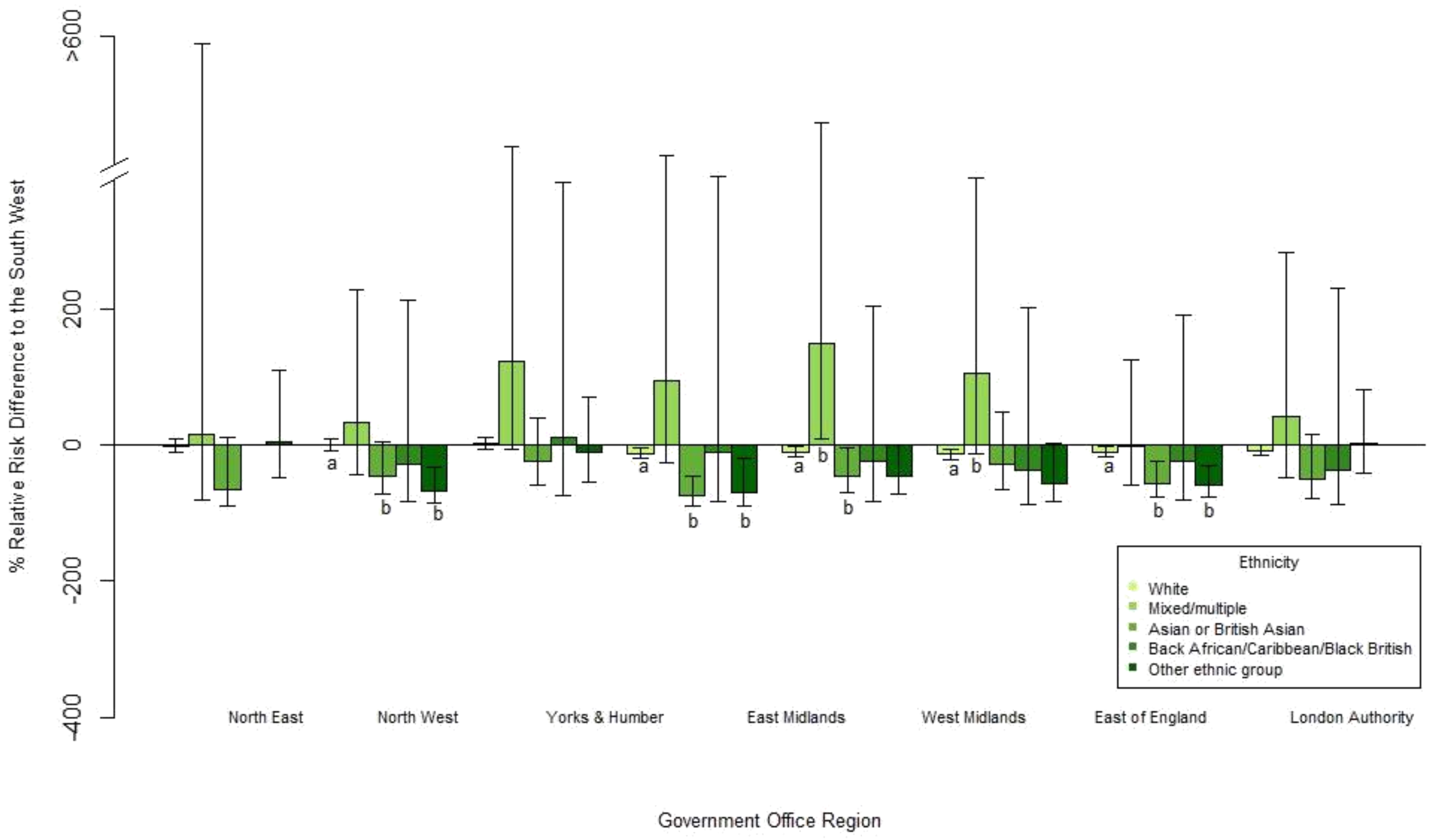

Supplementary Figure 7: Moderation effect of ethnicity on the association between Government Office Region and smoking

Note: Bars reflect 95% confidence intervals; Confidence intervals which do not straddle 0 indicate a significantly different risk of smoking in that region relative to the South West for the subgroup under investigation; ‘a’ and ‘b’ reflect a significant interaction effect at p<0.05 i.e. the relative risk difference of smoking for sub-group ‘a’ is significantly different to subgroup ‘b’

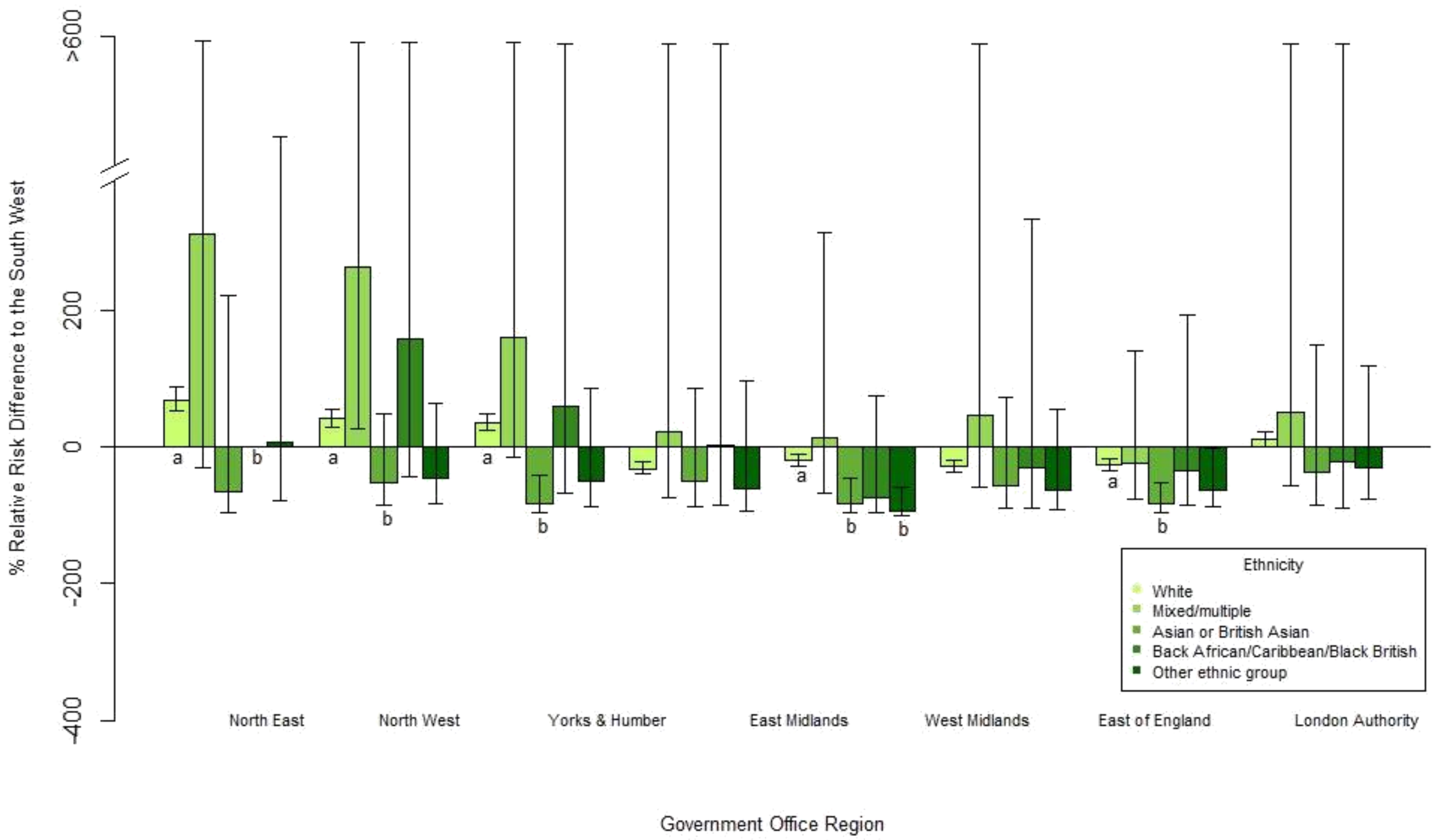

Supplementary Figure 8: Moderation effect of ethnicity on the association between Government Office Region and high-risk drinking

Note: Bars reflect 95% confidence intervals; Confidence intervals which do not straddle 0 indicate a significantly different risk of high-risk drinking in that region relative to the South West for the subgroup under investigation; 'a' and 'b' reflect a significant interaction effect at p<0.05 i.e. the relative risk difference of high-risk drinking for sub-group 'a' is significantly different to subgroup 'b'

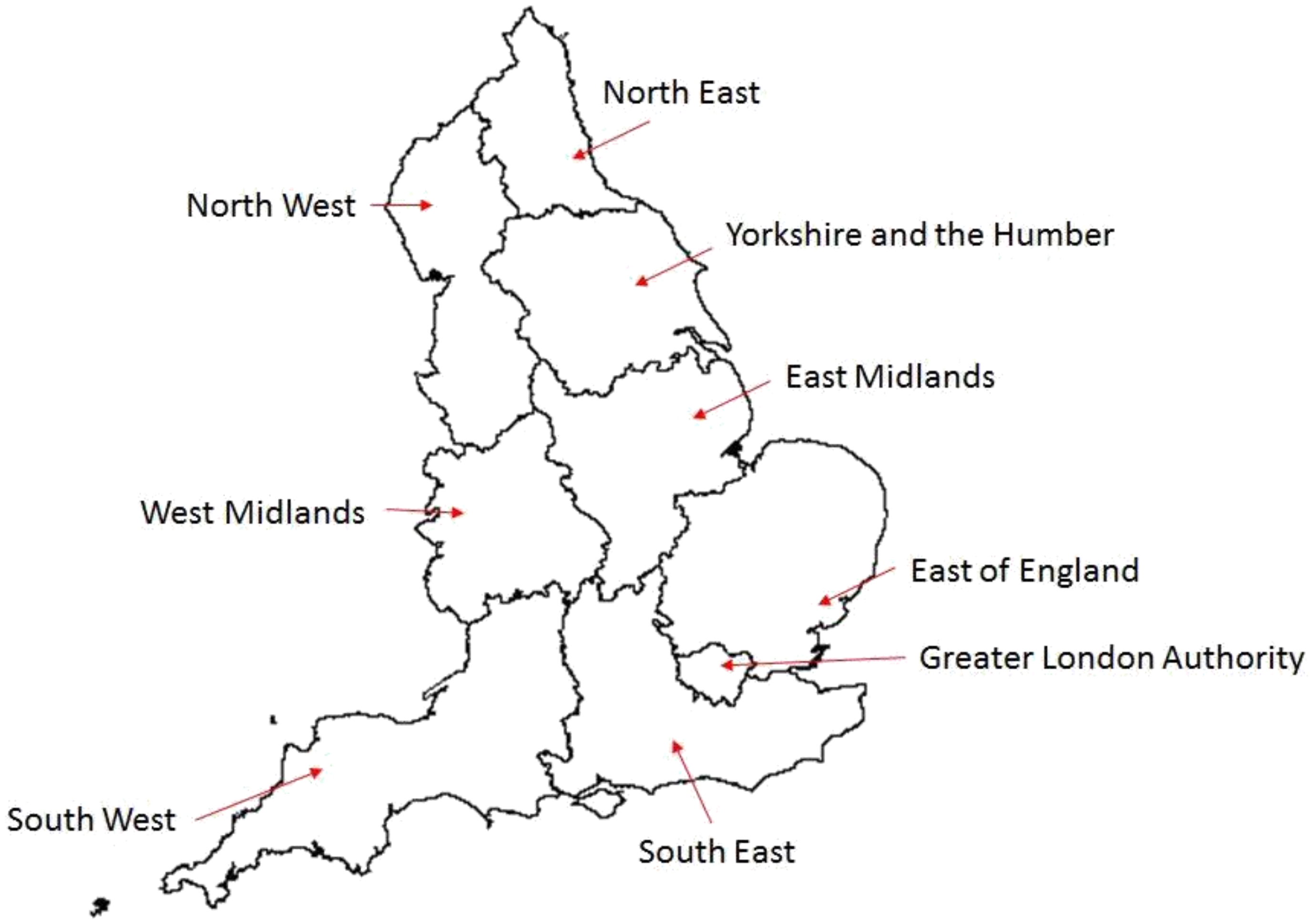

Supplementary Figure 9: Government Office Regions in England
